# Nomograms to predict outcome for patients undergoing venoarterial extracorporeal membrane oxygenation treatment for septic shock

**DOI:** 10.1007/s10047-025-01523-w

**Published:** 2025-08-26

**Authors:** Kunlin Hu, Jing Wei, Xinyu Chi, Jiwang Zhang, Xuanliang Zhao, Liqiu Lu, Yufeng Liao, Shulin Xiang, Bin Xiong

**Affiliations:** 1 Department of Critical Care Medicine, The People’s Hospital of Guangxi Zhuang Autonomous Region, No. 6, Taoyuan Road, Nanning, 530021 China; 2 Research Center of Communicable and Severe Diseases, The People’s Hospital of Guangxi Zhuang Autonomous Region (Guangxi Academy of Medical Sciences), Nanning, 530021 China

**Keywords:** Septic shock, Venoarterial extracorporeal membrane oxygenation, Mortality risk, Nomogram

## Abstract

Venoarterial extracorporeal membrane oxygenation (VA-ECMO) is increasingly being employed to treat patients with refractory septic shock. Despite its growing use, there is a notable absence of prognostic assessment tools specifically designed for septic shock patients who have received VA-ECMO therapy. The aim of this study is to develop and validate a prognostic model for patients with refractory septic shock undergoing VA-ECMO, and to identify those who may derive the greatest benefit from this treatment. This single-center, retrospective cohort study was conducted at a comprehensive intensive care unit in China. Adult patients with refractory septic shock who received VA-ECMO treatment were included. Two hundred consecutive patients were randomly divided into training and validation cohorts in a 7:3 ratio. Least absolute shrinkage and selection operator regression analysis was employed to select relevant variables for the logistic regression model, and its performance was tested in both training and validation cohorts based on discrimination, calibration, and net benefit. Between January 2019 and September 2024, 293 patients were screened, 200 of whom were eligible and were divided into development (*n* = 140) and validation (*n* = 60) cohorts. The 28-day survival rate was 23.0%, and median duration of ECMO run was 6.0 days (IQR 2.0–8.0). Age, APACHE II score at ICU admission, immunosuppression status, hypertension, IL-6, and APTT measured within 6 h before ECMO initiation were the six predictors included in the nomograms. The nomogram demonstrated strong discriminative power in the training cohort (area under the curve [AUC]: 0.873, 95% CI 0.812–0.929), as well as in the validation cohort (area under the curve [AUC]: 0.818 (95% CI 0.687–0.920). The model's reliability in predicting outcomes was evident from the high consistency between predicted probabilities and observed proportions during calibration. Decision curve analysis indicated that the model's clinical benefit was advantageous. The novel validated nomogram is designed to predict outcomes after VA-ECMO treatment in individuals with refractory septic shock. It can support physicians in performing precise mortality risk evaluations and making more informed decisions regarding the application of VA-ECMO treatment.

## Introduction

Septic shock represents a significant public health concern, characterized by elevated rates of mortality and morbidity [1]. The mortality rate of septic shock is up to 51.94%, while the mortality rate for refractory septic shock is 80–90% [2, 3]. Factors linked to sepsis mortality include age, heart failure, cancer, immunosuppression, elevated lactate levels, prothrombin time (PT), and infection sites [2, 4–6].

Venoarterial extracorporeal membrane oxygenation (VA-ECMO) may function as a bridge to recovery from septic shock; however, its efficacy remains uncertain. A meta-analysis of 468 refractory septic shock patients treated with VA-ECMO found higher survival rates in those with sepsis-induced cardiomyopathy [left ventricular ejection fraction (LVEF) < 35%] compared to those with normal cardiac function [7]. Conversely, other studies suggest early VA-ECMO use in distributive shock patients may enhance outcomes [8]. The 2021 guidelines did not offer specific recommendations concerning the use of VA-ECMO for the treatment of refractory septic shock [9]. ECMO is a resource-intensive intervention. Consequently, it is essential to identify key patient characteristics that are associated with an increased probability of survival to enhance the generalizability of VA-ECMO in cases of septic shock.

Several predictive models have been specifically developed for patients undergoing VA-ECMO [10]. Among these, the ENCOURAGE score is designed to assess the risk of mortality in patients receiving VA-ECMO following myocardial infarction [11], while the REMEMBER score predicts outcomes for patients undergoing VA-ECMO after coronary artery bypass grafting [12], the SAVE score is utilized to estimate survival rates in cases of refractory cardiogenic shock [13]. However, there is a lack of research on prognostic models for VA-ECMO specifically tailored to patients with septic shock. Consequently, by utilizing data from a cohort of septic shock patients who received VA-ECMO treatment, we aimed to develop and validate a prognostic model. This model has the potential to improve decision-making processes, facilitating the targeted application of VA-ECMO in patients with a higher likelihood of survival.

## Methods

### Study population

We conducted a single-center, retrospective study. The Institutional Review Board (IRB) of People's Hospital of Guangxi Zhuang Autonomous Region approved this study, the exemption and data management (approval number: IIT-2024-93). The study population comprised patients who received VA-ECMO for septic shock between January 1, 2019, and September 30, 2024, at the People's Hospital of Guangxi Zhuang Autonomous Region, in Guangxi, China. The diagnostic criteria for septic shock were based on the 2016 international guidelines [14]. All patients underwent a 6-h septic shock bundle treatment, including fluid resuscitation, vasopressors, antibiotics, and other regular interventions. The indication for initiating VA-ECMO was cardiogenic shock caused by septic cardiomyopathy (Cardiac Index, CI < 2.0), in cases where conventional vasoactive drugs are insufficient to maintain stability, defined as Vasoactive-Inotropic Score (VIS) > 150 points (calculated as [Dobutamine + (Norepinephrine + Epinephrine) × 100]) [15–18]. Patients who received hybrid mode (VAV or VVA mode), withdrawal of treatment, with underlying heart failure (LVEF < 35%) and pregnant women were excluded.

### Data collection

Variables including demographics, condition upon admission, clinicopathological data pre-ECMO and 28-day outcomes were collected. Demographic data included gender, age, and history of hypertension, diabetes mellitus, coronary heart disease (CHD), immunosuppression (patients who had undergone organ transplantation or hematopoietic stem cell transplantation, patients with solid tumors whose progression or remission period was less than 5 years, patients who received radiation therapy within the past 3 months or chemotherapy within the past 1 month, and patients who had been taking long-term or high-dose corticosteroids) [19]. Condition upon admission included APACHE II score and SOFA score. The pre-ECMO variables comprised cardiac arrest caused by septic cardiomyopathy and refractory shock requiring extracorporeal cardiopulmonary resuscitation (ECPR), body temperature, systolic blood pressure (SBP) and diastolic blood pressure (DBP), norepinephrine bolus (μg/kg min), epinephrine bolus (μg/kg min) within 6 h of cannulation, platelet count (PLT, 10^9^/L), pH, pO_2_ (mmHg), pCO_2_ (mmHg), lactic acid (mmol/L), albumin (ALB, g/L), N-terminal pro-B-type natriuretic peptide (NT-proBNP, pg/mL), procalcitonin (PCT), activated partial thromboplastin time (APTT, s), interleukin-6 (IL-6, pg/mL), left ventricular ejection fraction (LVEF) within 24 h before VA-ECMO.

### Statistical analysis

All analyses were conducted using R software, version 4.4.1. Continuous variables were summarized using means for normal distributions and medians otherwise, with normality assessed via the Shapiro–Wilk test. For continuous variables, comparisons were made using either Student's *t* test or the Wilcoxon signed-rank test, while categorical variables were compared using the chi-square test. The sample size was determined based on a ratio of 10 events per variable for logistic regression analysis [20]. The variables of procalcitonin and LVEF (measured by bedside echocardiography) with more than 20% missing values were removed [21, 22], and the remaining missing values were imputed using Multiple Imputation by Chained Equations (MICE) imputation. The variable IL-6 was scaled by dividing by 100 to mitigate the issue of excessively small centroid intervals arising from its large numerical values. A 7:3 ratio was used to randomly assign participants to a training set and an internal validation set. Stratified random sampling was conducted using the `createDataPartition` function from the `caret` package in R, to maintain consistent distribution proportions of categorical variables between the subsets and the original dataset. A fixed random seed was applied to ensure the reproducibility of the experiment. The training set was utilized for optimizing model parameters, whereas the independent validation set was used for the ultimate evaluation of the model's performance. Variables were screened using least absolute shrinkage and selection operator (LASSO) regression analysis. The variables identified through LASSO regression analysis were incorporated into a multivariate logistic regression model. Factors with *P* values < 0.1 were ultimately selected for inclusion in the predictive model. Subsequently, a nomogram was developed to estimate the risk of 28-day mortality. A bootstrapping validation procedure with 1000 iterations was applied to the training set. To evaluate model discrimination, the receiver operating characteristic (ROC) curve was utilized, while a calibration curve was produced to assess model calibration.

## Results

### Demographics and clinical characteristics

**Fig. 1:**
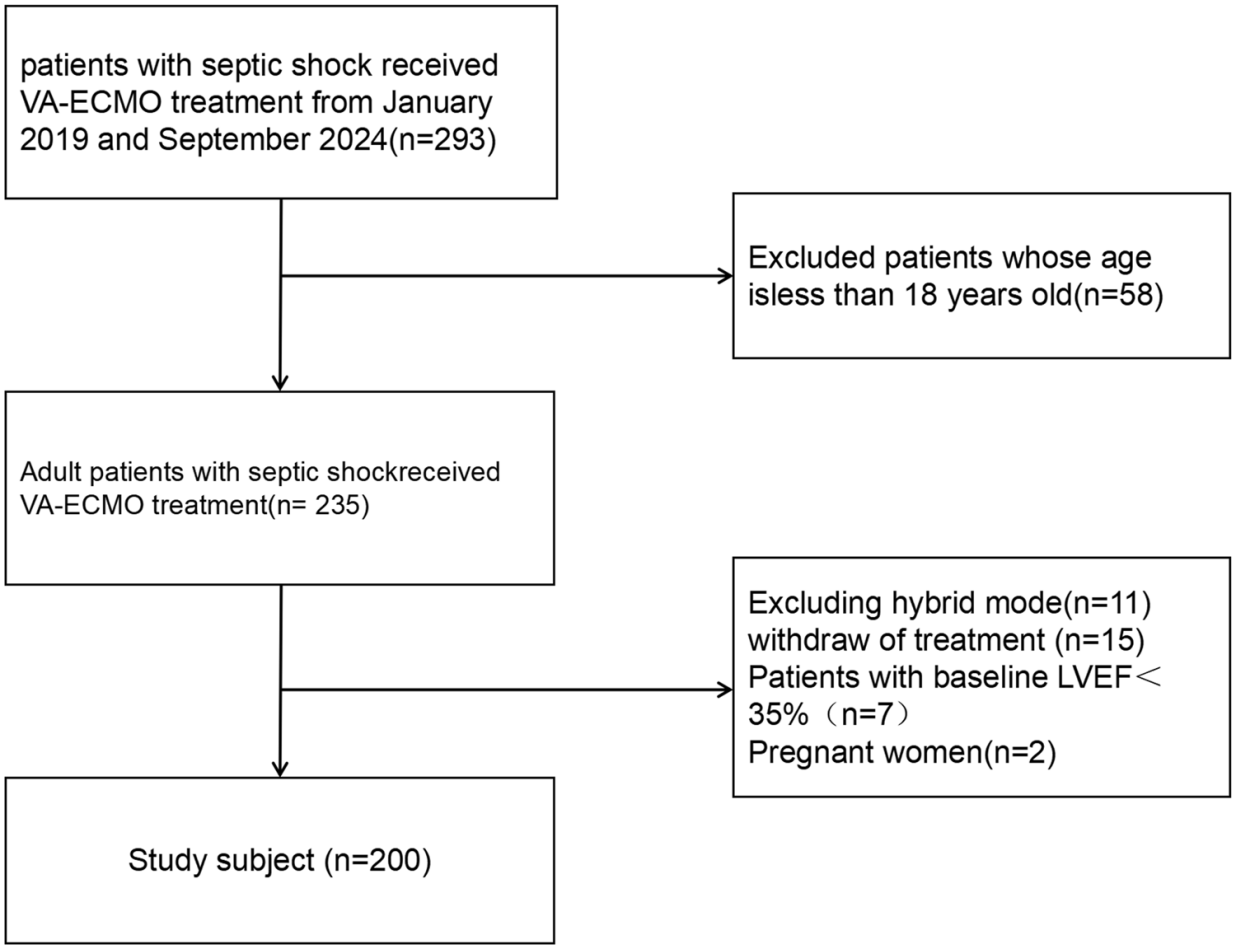
The flowchart showing the selection procedure

A total of 293 patients were treated with VA-ECMO for septic shock at the People's Hospital of Guangxi Zhuang Autonomous Region between January 2019 and June 2024. Of these, 58 patients who were younger than 18 years and 35 adult patients who did not meet the inclusion criteria were excluded (Fig. 1).

The two hundred patients included were 59.74 ± 16.53 years old and 72.0% were male. The most common underlying diseases were diabetes mellitus (T2DM; 30.0%) and hypertension (29.5%). On admission to the ICU, the patients had a mean APACHE II score of 31 ± 9 points and a mean SOFA score of 14 ± 4 points. The mean blood lactate level before ECMO initiation was 10 mmol/L. Before ECMO treatment, the mean dose of norepinephrine was 1.74 ± 0.60 µg/kg/min and the mean dose of epinephrine was 1.16 ± 0.84 µg/kg/min. The median duration of the ECMO support was 6.00 (2.00, 8.00) days. The median survival time was 9 (3, 22) days. The 28-day mortality rate and the 90-day mortality rate were 77.0% and 85.1%, respectively. Baseline characteristics and outcomes were well balanced between the two groups (as shown in Tables 1, 2).

**Table 1:** Demographics and clinical characteristics

Characteristics	Total (*n* = 200)	Training group (*n* = 140)	Test group (*n* = 60)	*P*
Age, mean ± SD	59.74 ± 16.53	59.74 ± 16.34	59.73 ± 17.13	0.99
Male, *n* (%)	144 (72.00)	96 (68.57)	48 (80.00)	0.10
T2DM, *n* (%)	60 (30.00)	47 (33.57)	13 (21.67)	0.09
Hypertension, *n* (%)	59 (29.50)	42 (30.00)	17 (28.33)	0.81
CHD, *n* (%)	50 (24.04)	34 (24.29)	13 (21.67)	0.69
Immunosuppression, *n* (%)	40 (20.00)	27 (19.29)	13 (21.67)	0.70
APACHEⅡII, mean ± SD	30.89 ± 9.00	30.98 ± 9.37	30.68 ± 8.56	0.83
SOFA, mean ± SD	14.00 ± 4.00	14.00 ± 4.00	13.00 ± 4.00	0.33
*Infection sources, n (%)*				
Pneumonia	152 (76.00)	105 (75.00)	47 (78.33)	0.82
Bloodstream infection	17 (8.50)	11 (8.20)	6 (10.00)	0.65
Urinary tract infection	11 (5.50)	8 (5.70)	3 (7.50)	0.72
Abdominal infection	30 (15.00)	20 (14.29)	10 (16.67)	0.62
Skin infection	12 (6.00)	7 (5.00)	5 (8.33)	0.75
*Hemodynamic status (mean ± SD)*				
Systolic pressure, mmHg	90.00 ± 35.00	91.06 ± 36.38	89.90 ± 35.22	0.83
Diastolic pressure, mmHg	51.00 ± 22.00	51.00 ± 21.00	52.25 ± 23.34	0.66
Norepinephrine, μg/kg min	1.74 ± 0.60	1.81 ± 0.58	1.60 ± 0.60	0.02
Epinephrine, μg/kg min	1.16 ± 0.84	1.19 ± 0.85	1.09 ± 0.80	0.42
VIS score	280 ± 15	300 ± 14	269 ± 14	0.45
ECPR, *n* (%)	30 (15.00)	19 (13.57)	11 (27.50)	0.39
*Pre-ECMO clinical status (mean ± SD)*				
PLT, 10^9^/L	99.66 ± 93.58	104.02 ± 91.63	89.46 ± 98.00	0.32
pH	7.29 ± 0.16	7.30 ± 0.17	7.28 ± 0.16	0.35
pO_2_, mmHg	124.99 ± 89.91	132.97 ± 93.07	106.37 ± 79.73	0.06
pCO_2_, mmHg	41.41 ± 21.56	38.87 ± 17.78	47.35 ± 27.78	0.03
Lac, mmol/L	10.08 ± 6.38	10.40 ± 6.45	9.34 ± 6.18	0.29
ALB, g/L	25.59 ± 7.13	25.64 ± 7.20	25.46 ± 7.00	0.87
NT-proBNP, pg/ml	14,665.44 ± 13,028.66	15,322.28 ± 13,524.62	13,132.82 ± 11,755.17	0.25
APTT, s	78.57 ± 48.64	77.97 ± 41.52	79.97 ± 48.57	0.77
IL-6, 100 pg/ml	24.773 ± 21.533	25.377 ± 21.565	23.364 ± 21.572	0.55

T2DM, diabetes mellitus; CHD, coronary heart disease; ECPR, extracorporeal cardiopulmonary resuscitation; PLT, platelet; pO_2_, partial pressure of oxygen; pCO2, partial pressure of carbon dioxide; lac, lactic acid; ALB, albumin; NT-proBNP, N-terminal pro-B-type natriuretic peptide; APTT, activated partial thromboplastin time; IL-6, interleukin-6. VIS score: Vasoactive-Inotropic Score = dobutamine (μg/kg min) + norepinephrine (μg/kg min) × 100 + epinephrine (μg/kg min) × 100

**Table 2:** Clinical outcomes

Clinical outcome	Total (*n* = 200)	Training group (*n* = 140)	Test group (*n* = 60)	*P*
VA-ECMO support duration (day)	6.00 (2.00, 8.00)	6.00 (2.00, 8.00)	5.00 (2.00, 8.00)	0.82
Successful weaning, *n* (%)	74 (37.00)	52 (37.14)	22 (36.66)	0.25
28-day survival, *n* (%)	46 (23.00)	31 (22.14)	15 (25.00)	0.66
Hospital mortality, *n* (%)	170 (85.00)	120 (85.71)	50 (83.33)	0.45
Total survival period (day)	9 (3, 22)	10 (3, 25)	8 (3, 22)	0.37
Survival period of survival	375 (95, 705)	368 (90, 768)	465 (100, 1281)	0.12
Survival period of non-survivals	6 (3, 12)	5 (3, 10)	8 (4, 16)	0.34
Cause of death, *n* (%)				
Septic shock (primary disease)	123 (72.35)	86 (71.67)	37 (74.00)	0.59
MODS	40 (23.53)	29 (24.17)	11 (22.00)	0.09
Neurological disorder	4 (2.35)	3 (2.50)	1 (2.00)	0.88
Bleeding	3 (1.76)	2 (1.67)	1 (2.00)	0.79
*VA ECMO-related complications*				
Neurological complications	8 (4.00)	6 (4.29)	2 (3.33)	0.51
Bleeding	12 (6.00)	9 (6.43)	3 (5.00)	0.68
Lower limb ischemia	7 (3.50)	5 (3.57)	2 (3.33)	0.15

MODS: multiple organ dysfunction syndrome; MODS: multiple organ dysfunction syndrome; neurological complications: cerebral infarction and intracerebral hemorrhage; bleeding: gastrointestinal bleeding, retroperitoneal hematoma, pulmonary hemorrhage and mucocutaneous hemorrhage

### Model establishment and evaluation

**Fig. 2:**
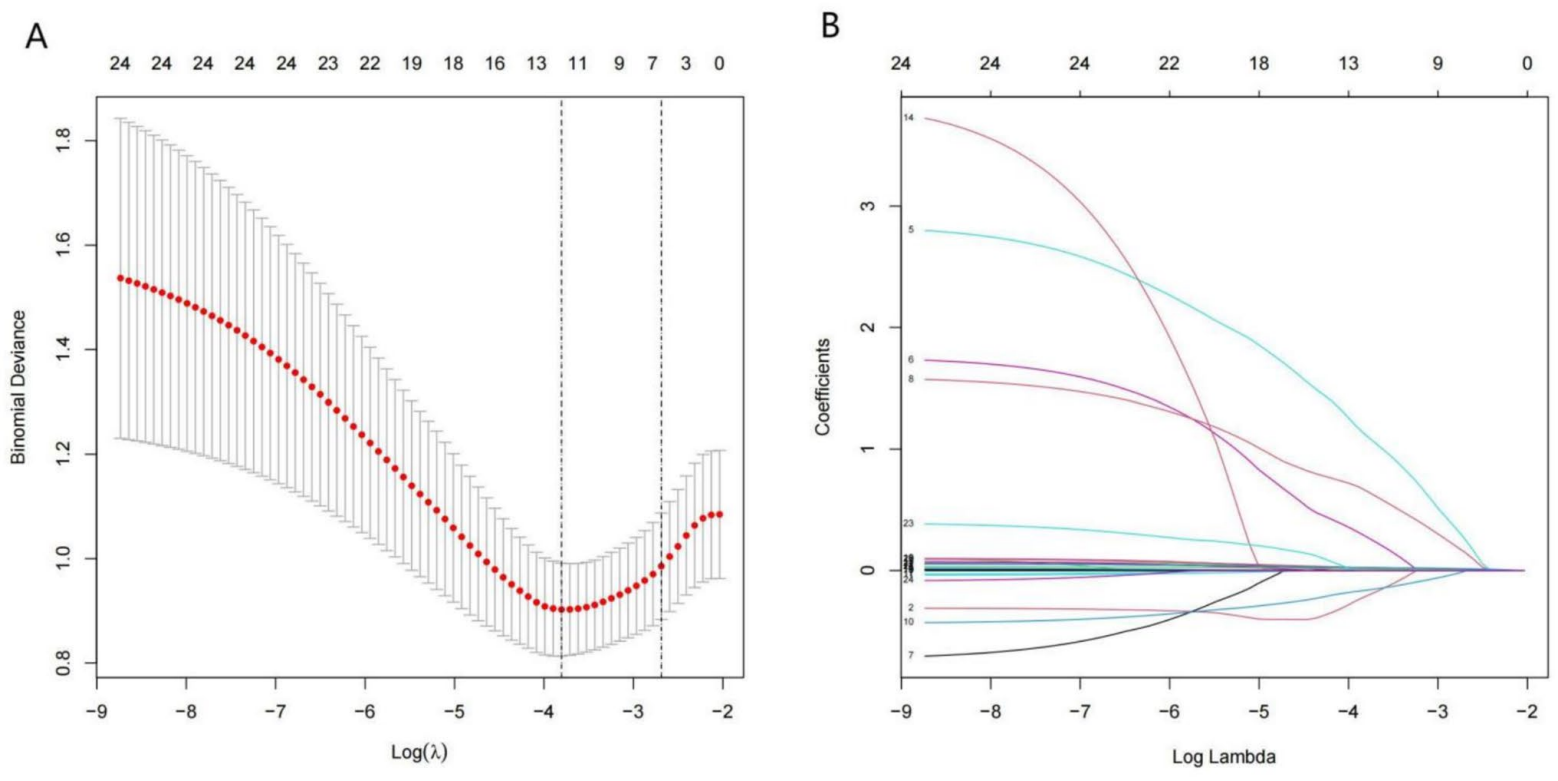
Variable selection: LASSO multiple logistic regression model. **A** Upon verifying the optimal parameter in the LASSO model, we plotted a partial likelihood deviation curve (binomial deviation) and pair number, and marked a vertical dashed line at 1 SE. **B** Twelve variables with nonzero coefficients were identified by finding the best

A total of 24 variables were included in the LASSO regression. Eleven variables identified by the LASSO-Min model (Fig. 2) were integrated into the multivariate logistic regression analysis.

Six variables with a *P* value < 0.1 were ultimately chosen for the predictive model: age, APACHE II score at admission, immunosuppression status, hypertension, IL-6, and APTT measured within six hours before ECMO initiation (Table 3). Age and IL-6 emerged as independent prognostic risk factors for refractory septic shock treated with VA-ECMO (*P* < 0.05). Sensitivity analysis demonstrated superior discrimination (AUC 0.873 vs. 0.774) and calibration for the model incorporating variables with *P* < 0.1 compared to those with *P* < 0.05, supporting the adoption of this six-variable model.

**Table 3:** Analysis of mortality risk using multivariate logistic regression

Variable	OR (95% CI)	*P* value
Age	1.04 (1.00–1.07)	0.042*
Gender	0.51 (0.14–1.68)	0.282
APACHEⅡ II score	1.06 (0.99–1.13)	0.082*
Immunosuppression	8.05 (1.14–177.03)	0.081*
Extracorporeal cardiopulmonary resuscitation	2.41 (0.32–52.09)	0.462
Hypertension	3.71 (0.91–19.68)	0.087*
Body temperature	0.74 (0.46–1.16)	0.202
Systolic blood pressure	1.00 (0.98–1.02)	0.859
PT	1.07 (1.00–1.20)	0.112
APTT	1.02 (1.00–1.05)	0.054*
IL-6 (100 pg/mL)	1.03 (1.01–1.06)	0.02*

**Fig. 3:**
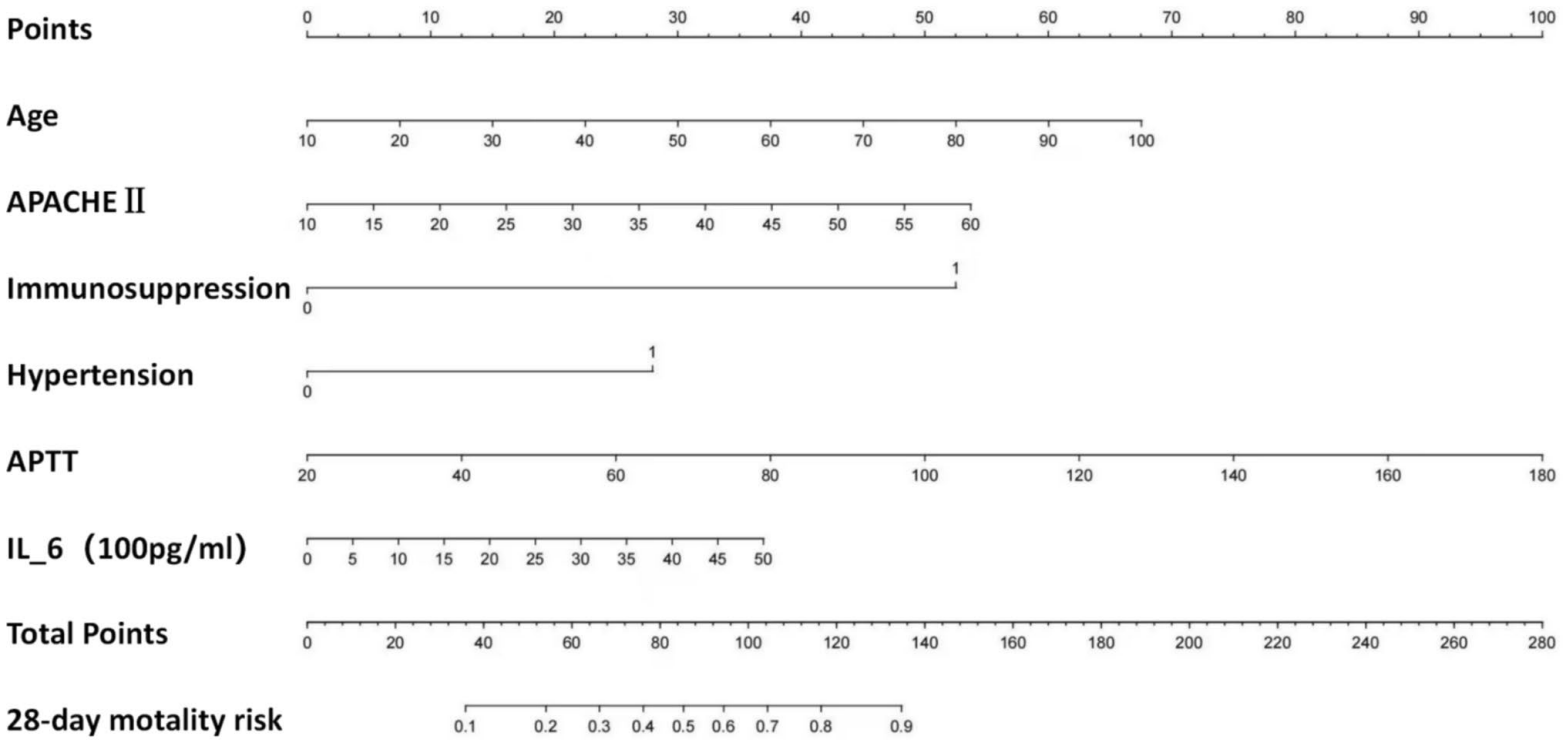
Nomogram based on age, APACHE II score at admission, immunosuppression status, hypertension, IL-6 and APTT measured within six hours prior to ECMO initiation in predicting the 28-day mortality risk of septic shock patients. The scale ruler values for each variable matched their points on the first line. These points were summarized as total points, which indicated a patient's 28-day probability of mortality. The overall score was determined by summing the individual scores of each variable presented in the nomogram. Each variable was assessed for the patient, and the total score was assigned based on the nomogram

Logistic regression was employed to develop a nomogram aimed at predicting the risk of 28-day mortality for septic patients undergoing VA-ECMO treatment (Fig. 3).

**Fig. 4:**
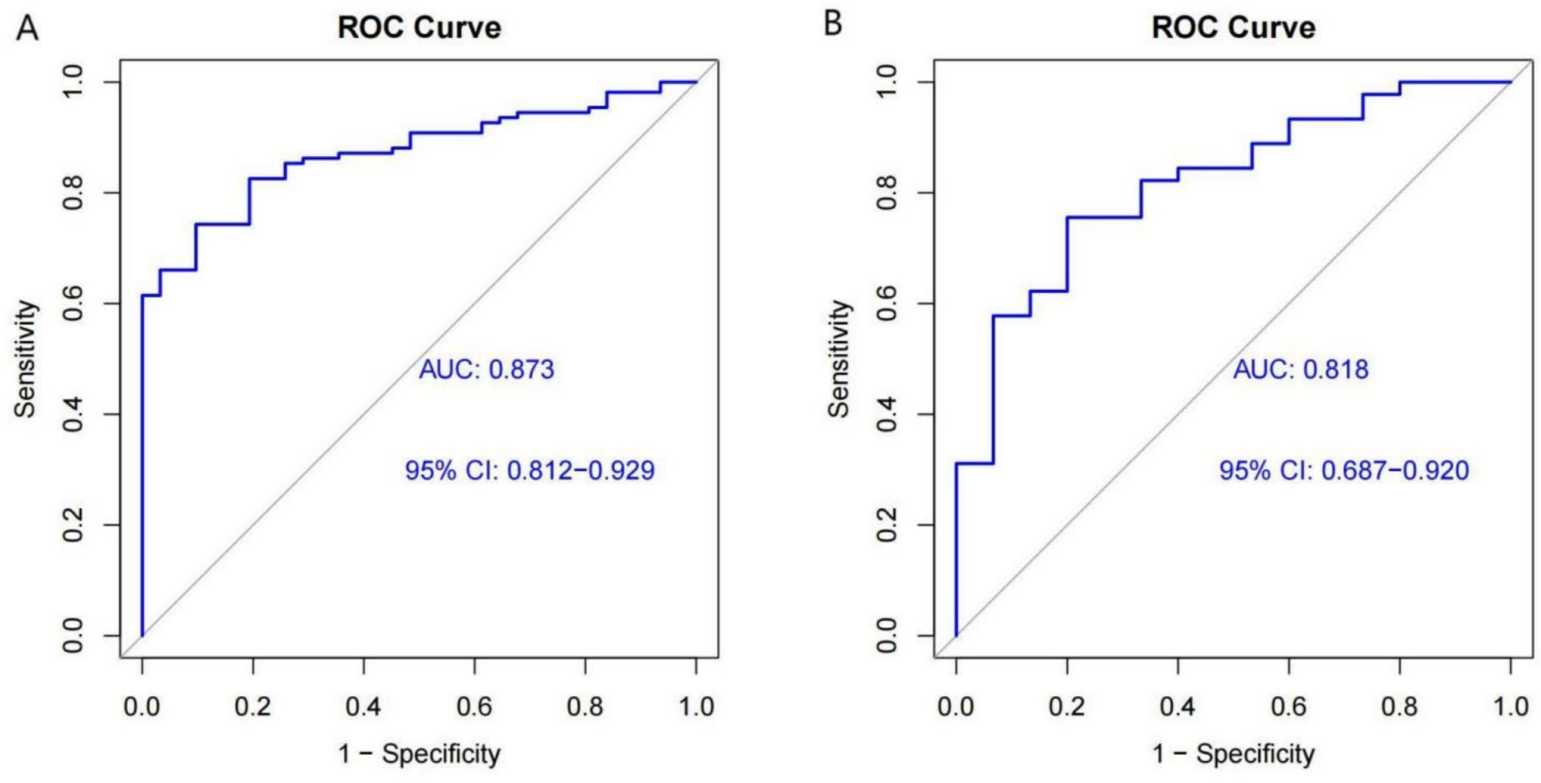
ROC curve analysis of the training cohort (**A**) and the test cohort (**B**). ROC curve of the training cohort is 0.873 (95% CI 0.812–0929), ROC curve of the test cohort is 0.818 (95% CI 0.687–0920)

The area under the curve (AUC) of the nomogram was 0.873 for the training dataset and 0.818 for the validation dataset (Fig. 4).

**Fig. 5:**
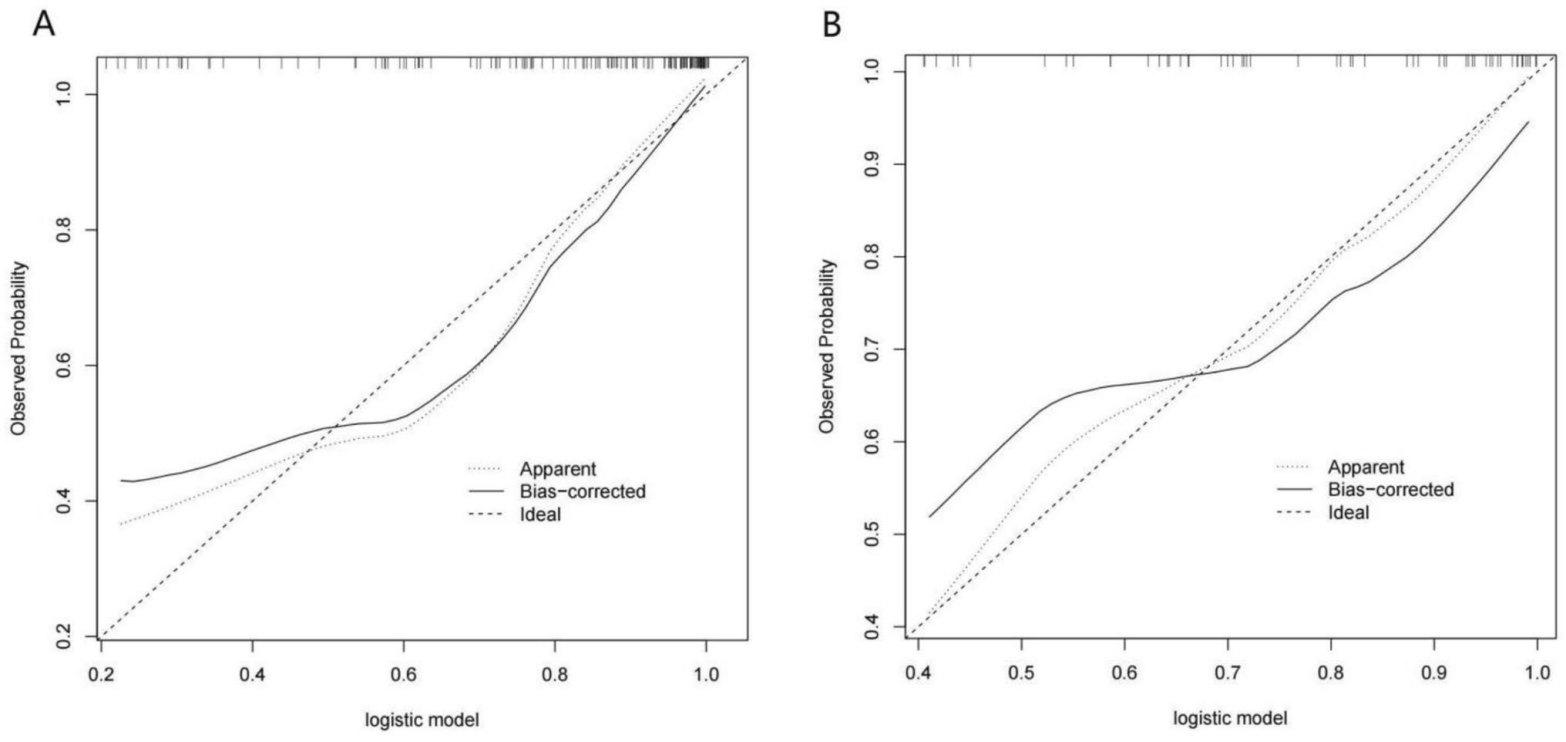
Calibration curve of the logistic model in the training (**A**) and testing (**B**) datasets. The *x*-axis represents the nomogram-predicted mortality risk, and the *y*-axis represents the actual mortality. The diagonal dotted line denotes the ideal prediction by a perfect model. The line labeled "Apparent," illustrates the performance of the model trained on the original dataset. In contrast, the black line labeled "Bias-corrected" depicts the performance of the model following repeated bootstrapping of the samples, which addresses overfitting. The calibration curves demonstrated that the model exhibited a high degree of calibration accuracy

The calibration curves for the model predicting 28-day mortality demonstrated strong consistency between predicted and observed outcomes in both the training and validation sets (Fig. 5).

### Clinical use of the nomogram

**Fig. 6:**
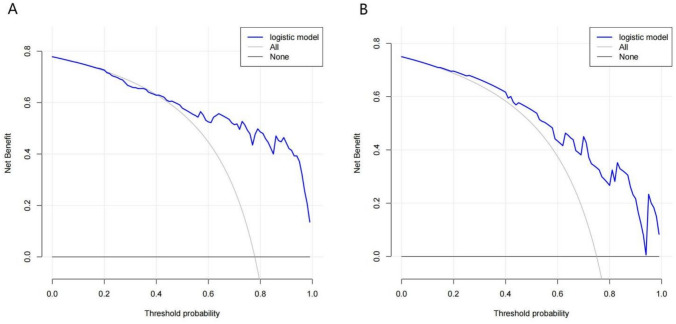
Decision curve analysis of the nomogram in the training set (**A**) and the testing set (**B**). The *y*-axis quantifies the net benefit. The blue line illustrates the nomogram, while the gray line depicts the assumption that all patients received ECMO treatment. Conversely, the black line represents the assumption that no patients received ECMO treatment. The model demonstrates clinical significance when the patient's risk of mortality surpasses 16%

Decision curve analysis (DCA) further confirmed the predictive efficacy of the nomogram for 28-day mortality. The decision curve analysis showed that predicting 28-day mortality in septic shock patients undergoing VA-ECMO treatment provided greater net benefit compared to alternative strategies when the threshold probability exceeded 16% (Fig. 6).

## Discussion

VA-ECMO is employed in the management of patients experiencing severe septic shock, despite the high mortality and complication rates associated with its use, as well as the socio-economic burdens arising from the need for specialized critical care [7]. Therefore, it is imperative to prioritize VA-ECMO for patients who are most likely to derive benefit from the intervention. Previous studies have proposed several models for predicting mortality risk following VA-ECMO in cases of refractory cardiogenic shock. However, none of these models have been specifically developed for septic shock [10–13]. In this single-center, retrospective study, a novel and practical nomogram was developed to predict 28-day mortality risk following VA-ECMO treatment for refractory septic shock. To the best of our knowledge, this is the first nomogram to predict outcomes in patients with septic shock undergoing VA-ECMO. This model is designed to aid physicians in performing accurate risk assessments and making informed decisions regarding the initiation of VA-ECMO treatment. Clinicians can utilize this model to predict mortality risk in patients suffering from refractory septic shock prior to VA-ECMO therapy. For instance, a 61-year-old patient with a medical history of hypertension and no evidence of immunosuppression was admitted with an APACHE II score of 37. Prior to the initiation of extracorporeal membrane oxygenation (ECMO), the patient's IL-6 level was 163.6 pg/mL, and the APTT was 46.8 s. The total score was calculated as 102 points, corresponding to a mortality risk of 70%. The model predicted that the patient had a 70% risk of mortality despite ECMO therapy. A high predicted risk suggests that ECMO may offer little advantage, while a lower risk indicates potential benefit, justifying ECMO initiation. Furthermore, comprehensive communication with family members regarding ECMO initiation is essential. As families primarily focus on patient survival prospects, this model enables clinicians to effectively convey mortality risks. Additionally, it offers an evidence-based framework to facilitate family participation in decisions about ECMO initiation.

A meta-analysis including 14 studies with 468 patients demonstrated that the overall survival rate for patients with septic shock undergoing VA-ECMO is 36.4% (ranging from 15 to 71%) [23]. The mortality rates reported in this study are consistent with those observed in previous research. The patients’ condition is exceedingly critical, as evidenced by elevated APACHE II of 31 and SOFA of 14 scores, pre-ECMO lactate levels of 10 mmol/L, consequently, the mortality rate is significantly high. The mortality rate for septic shock patients on VA-ECMO is affected by both patient selection and the timing of ECMO initiation [24]. Our study found that although our center set ECMO initiation criteria at a cardiac index (CI) below 2.0 and a vasoactive-inotropic score (VIS) over 150, the actual mean VIS at initiation was 280 ± 15, indicating significant delays. This delay led to prolonged organ ischemia and hypoxia, causing multiple organ failure and irreversible decline. Evidence on the best timing for VA-ECMO in sepsis is scarce. A meta-analysis by Ling et al. showed a 23.4-h average from septic shock diagnosis to ECMO initiation, with a 36.4% survival rate [7]. Cheng et al. found that starting ECMO within 96 h improved mortality outcomes [16]. Delays at our center are partly due to patients arriving in critical condition from other wards or hospitals (typically exceeds 48 h). Furthermore, although our ECMO team manages 350–400 cases each year, it remains understaffed. In addition to the previously mentioned factors, it is essential to consider fatalities resulting from inflammatory response injuries and complications associated with ECMO [24]. Nevertheless, our predictive model is designed to evaluate mortality risk prior to the initiation of ECMO, and therefore, factors occurring post-initiation were not included in the analysis.

In this study, a total of 11 predictors, including demographic information, underlying medical conditions, severity upon admission and the situation before initiating VA-ECMO, were evaluated to reduce the bias associated with nomograms. LASSO regression analysis was employed to screen these variables, resulting in the identification of six key predictors of mortality risk. Among these factors, age serves as an independent risk determinant for mortality in patients with septic shock undergoing VA-ECMO therapy. This finding aligns with the results of the previous study [25]. Additionally, numerous studies have demonstrated that age is a critical predictor of mortality in septic shock [26–28] and patients undergoing VA-ECMO [13, 29–31].

Another independent risk factor identified was the level of IL-6 (100 pg/mL), marking the first report of its association with mortality in patients with septic shock receiving VA-ECMO treatment. IL-6 is a multifunctional glycoprotein produced by a variety of immune cells, including monocytes/ macrophages and T cells [32]. It holds significant clinical importance in evaluating disease severity and prognostic outcomes in sepsis and septic shock [33–36]. A study by Song et al. identified IL-6 as an independent risk factor for 28-day mortality from sepsis. Risnes et al. found that patients with higher levels of IL-6 undergoing cardiac surgery and receiving ECMO therapy had lower survival rates, and IL-6 could serve as a prognostic biomarker for survival in these patients [36]. IL-6 plays a critical role in the initiation and amplification of the inflammatory response in patients with sepsis [37]. It activates the coagulation pathway [38] and contributes to sepsis-induced capillary leakage and organ dysfunction [39]. In our study, IL-6 levels in patients with refractory septic shock were markedly elevated compared to those documented in existing literature [40, 41], indicating severe systemic inflammatory responses and a poor prognosis. To elucidate its role within the model, a 100-fold scaling was applied. In summary, IL-6 levels are significantly correlated with the prognosis of patients with septic shock undergoing VA-ECMO therapy. Therefore, assessing of a patient's IL-6 levels should be an integral factor for determining the appropriateness of initiating an ECMO protocol.

The model includes four covariates—immunosuppression status, hypertension, APACHE II score, and APTT—that are linked to septic shock and the prognosis of patients on ECMO, aligning with earlier findings [10, 42–44]. The model integrates six variables that are easily accessible through clinical methods, providing a substantial benefit for its broad implementation in clinical practice. To our knowledge, this work constitutes the most extensive single-center study to predict the prognosis of patients with septic shock who have received VA-ECMO therapy. It demonstrates excellent discrimination and calibration, alongside notable advantages in clinical application. Furthermore, the use of a nomogram improves the conciseness and visual clarity of the results [45–47], thereby highlighting its strong clinical applicability.

Our study has a few limitations. First, owing to the retrospective design of this study, the inclusion of PCT and LVEF was precluded by the substantial amount of missing data. Another rationale for excluding LVEF is that in critical clinical scenarios, the quantitative assessment of LVEF frequently necessitates reliance on eyeballing estimation as a practical alternative. Echocardiographic measurements may exhibit reduced reliability during episodes of sepsis due to the dynamic fluctuations in hemodynamic loading conditions, which can be exacerbated by interventions such as fluid resuscitation that affect preload and afterload [24]. Furthermore, the application of this model has been restricted to patients experiencing refractory shock associated with septic cardiomyopathy. Second, given that this was a single-center study, the findings may not be generalizable to other populations or applicable in different countries. It is recommended that future research involve multicenter studies for external or subgroup validation of the model. Third, immunosuppression was included in the final model notwithstanding the presence of an exceptionally wide confidence interval, further research involving larger cohorts is necessary to elucidate this relationship.

## Conclusions

In summary, a novel nomogram has been developed and validated to effectively predict the prognosis of patients with refractory septic shock treated with VA-ECMO. This tool will aid ICU clinicians in identifying patients most likely to gain survival benefits from VA-ECMO. Our study corroborates the utility of IL-6 as a prognostic biomarker, and the inclusion of six readily accessible clinical variables enhances the model's practical applicability. Additional validation studies are needed to test and refine the nomogram.

## Data Availability

Due to privacy and ethical restrictions, the data supporting the findings of this study cannot be made publicly available. However, the data can be provided upon reasonable request, requests should be directed to the corresponding author at xiangshulin27@163.com.
